# Efficient contour-based annotation by iterative deep learning for organ segmentation from volumetric medical images

**DOI:** 10.1007/s11548-022-02730-z

**Published:** 2022-09-01

**Authors:** Mingrui Zhuang, Zhonghua Chen, Hongkai Wang, Hong Tang, Jiang He, Bobo Qin, Yuxin Yang, Xiaoxian Jin, Mengzhu Yu, Baitao Jin, Taijing Li, Lauri Kettunen

**Affiliations:** 1 School of Biomedical Engineering, Faculty of Electronic Information and Electrical Engineering, Dalian University of Technology, Dalian, China; 2 Faculty of Information Technology, University of Jyväskylä, Jyväskylä, Finland; 3 Liaoning Key Laboratory of Integrated Circuit and Biomedical Electronic System, Dalian, China

**Keywords:** Medical image annotation, Deep learning, Organ segmentation, Interactive segmentation

## Abstract

**Purpose:**

Training deep neural networks usually require a large number of human-annotated data. For organ segmentation from volumetric medical images, human annotation is tedious and inefficient. To save human labour and to accelerate the training process, the strategy of annotation by iterative deep learning recently becomes popular in the research community. However, due to the lack of domain knowledge or efficient human-interaction tools, the current AID methods still suffer from long training time and high annotation burden.

**Methods:**

We develop a contour-based annotation by iterative deep learning (AID) algorithm which uses boundary representation instead of voxel labels to incorporate high-level organ shape knowledge. We propose a contour segmentation network with a multi-scale feature extraction backbone to improve the boundary detection accuracy. We also developed a contour-based human-intervention method to facilitate easy adjustments of organ boundaries. By combining the contour-based segmentation network and the contour-adjustment intervention method, our algorithm achieves fast few-shot learning and efficient human proofreading.

**Results:**

For validation, two human operators independently annotated four abdominal organs in computed tomography (CT) images using our method and two compared methods, i.e. a traditional contour-interpolation method and a state-of-the-art (SOTA) convolutional network (CNN) method based on voxel label representation. Compared to these methods, our approach considerably saved annotation time and reduced inter-rater variabilities. Our contour detection network also outperforms the SOTA nnU-Net in producing anatomically plausible organ shape with only a small training set.

**Conclusion:**

Taking advantage of the boundary shape prior and the contour representation, our method is more efficient, more accurate and less prone to inter-operator variability than the SOTA AID methods for organ segmentation from volumetric medical images. The good shape learning ability and flexible boundary adjustment function make it suitable for fast annotation of organ structures with regular shape.

**Supplementary Information:**

The online version contains supplementary material available at 10.1007/s11548-022-02730-z.

## Introduction

Nowadays, deep learning (DL) demonstrated promising performance for medical image analysis, and deep neural networks became the mainstream method for organ segmentation from medical images. So far, the training of an effective deep segmentation network still requires the annotation of large datasets. It is well known that manual annotation of volumetric medical images is extremely tedious and prone to subjective variabilities. Although some recent efforts have been made on few-shot or unsupervised learning, these methods are still not generalizable enough for different imaging modalities or target organs. Human supervision is still indispensable for most cases of deep segmentation network training. Therefore, efficient network training and human annotation methodologies are needed to relieve the burden of data annotation.

To tackle this problem, the strategy of annotation by iterative deep learning (AID) [1] becomes popular in the research community. In a typical AID workflow, a segmentation network is preliminarily trained via a small number of manually annotated data. Then, the preliminarily trained network is used for automatically annotating more training data. Since the preliminary trained network is not accurate enough, human supervisors are involved to proofread the automatic annotation results, and the human-corrected annotations are added to the training set to retrain a more accurate network. As this procedure is iteratively repeated, the network performance is gradually improved, and thus less-and-less user proofreading is needed.

The efficiency of an AID workflow mainly depends on two factors, i.e. the learning speed of the network model and the convenience of the proofreading method. On the one hand, a network with good learning ability can quickly approach satisfactory performance with a small number of training data. On the other hand, a convenient human-intervention method is essential for saving the interaction time and reducing the inter-operator variability. By far, many efforts have been made to improve the network learning ability. Methods based on few-shot [2–4] or semi-supervised learning [5–8] were proposed to train a segmentation network with limited training data. To alleviate the high annotation cost, weakly supervision approaches [9–11] were developed to complement the pixel-level full supervision. As for human-proofreading, there are already several publicly available medical image processing software with manual annotation main tools, such as ITK-SNAP [12], MITK [13], 3D Slicer [14], TurgleSeg [15] and Seg3D [16]. Most of them provided interaction functions including pixel painting, contour interpolation [13, 15, 17, 18], interactive level sets [19], surface adjustment [20], super-pixel [21] and super-voxel [22] modification. However, these tools were developed for general segmentation purposes; none of them was dedicatedly designed for efficient correction of neural network outputs. Recently, some specialized proofreading methods based on convolutional neural networks (CNNs) were proposed to involve human intervention into DL-based segmentation pipelines, such as DeepIGeoS [23], IFSeg [24] and BIFSeg [25]. These methods receive the user’s proofreading along with the medical image as the network inputs. For 3D segmentation networks, the speed of network inference cannot reach real-time output, limiting their applicability for efficient proofreading.

**Fig. 1:**
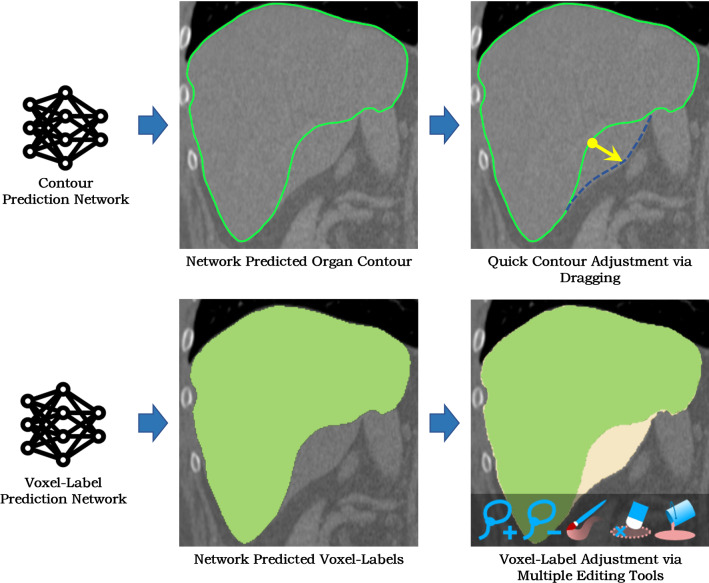
Comparison of contour representation and voxel-label-based representations of organ segmentation results. Contour representation facilitates direct incorporation of shape knowledge and convenient expert-correction. In contrast, voxel-label-based representation requires multiple editing tools for label correction

In this study, we further accelerate of AID workflow for organ segmentation from volumetric medical images. Our key idea is to use contour-based representation instead of voxel-label representation to improve the network learning ability and the user-interaction efficiency. By using contour representations, shape *prior* of the target organ is incorporated into the learning process, achieving anatomically plausible segmentation with only a few training images. Our user-interaction method also takes advantage of the contour-representation to facilitate real-time expert proofreading. As shown in Fig. 1, expert-proofreading of organ contours can be achieved via simple mouse interaction (e.g. dragging), while conventional voxel-label-based representation requires multiple manual editing tools such as pens, brushes and erasers. Our method is dedicated to the segmentation of human organs with strong shape characteristics. The source code and the proposed AID model are integrated into our open-source software AnatomySketch for the research community to use (https://github.com/DlutMedimgGroup/AnatomySketch-Software/tree/master/AID).

## Materials and methods

**Fig. 2:**
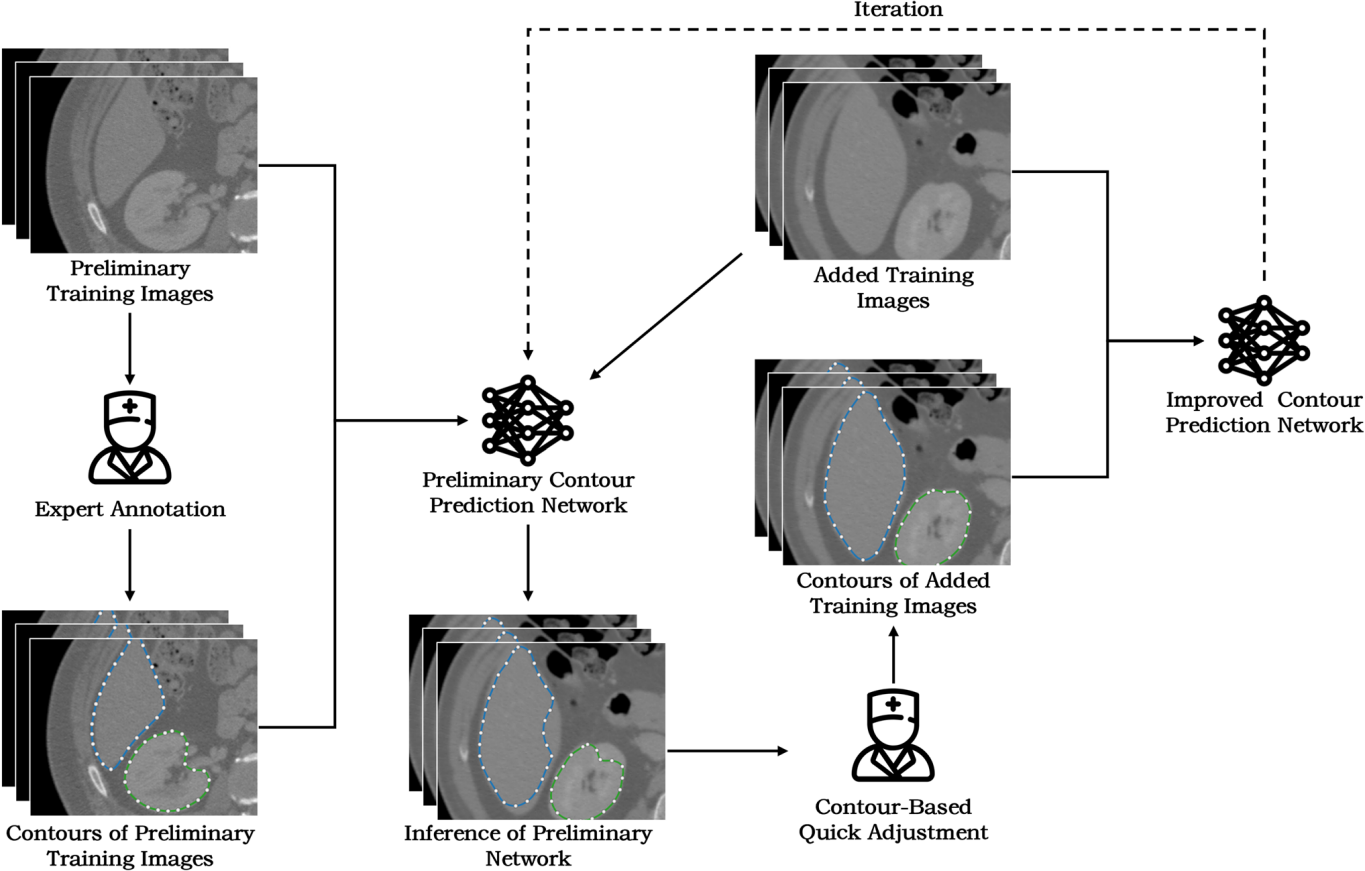
Workflow of our contour-based AID method

Our contour-based AID method is composed of an organ contour prediction network and an organ boundary adjustment module (as illustrated in Fig. 2). The learning loop starts from only a few expert-labelled preliminary training images. We convert the voxel labels into boundary contours to better represent the organ shape. Although the number of the preliminary training image is small (usually less than ten), our network can learn strong organ shape knowledge from the contour representation. The preliminarily learned shape knowledge helps the network to yield anatomically plausible segmentation of more training images, and the human expert quickly proofread the segmentation results using our boundary adjustment method. Then, the proofreading results are added to the training set and the network is retrained to achieve better prediction accuracy. The key idea of our method is to use contour representation instead of conventional label representation to facilitate better shape learning and faster expert proofreading. Details of the contour prediction network and the boundary adjustment method are described in the following sections.

### The contour prediction network

**Fig. 3:**
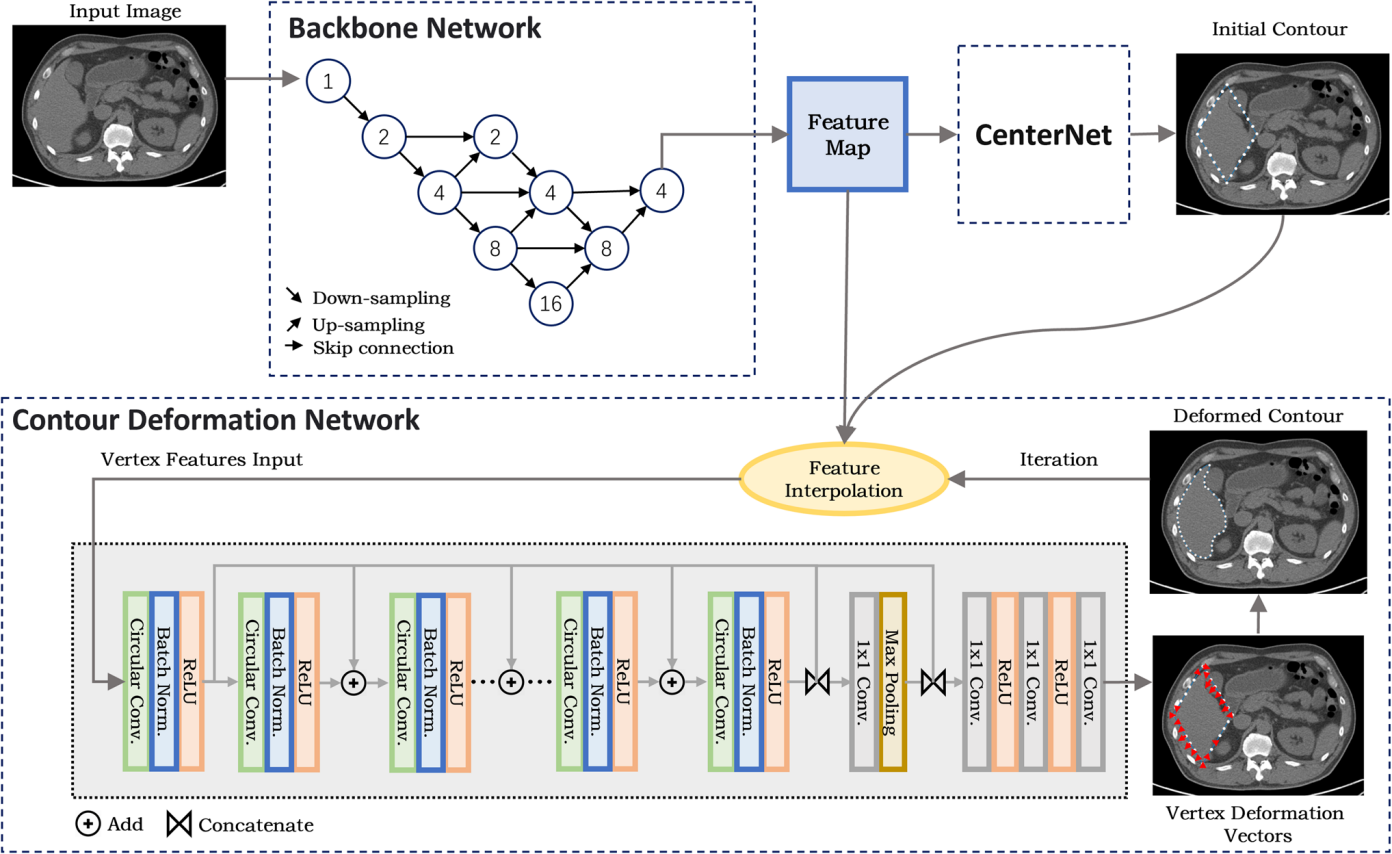
The architecture of the automated contour generation network. A multi-scale feature extraction backbone is used to extract features from the image. The feature map is fed into the CenterNet structure to obtain the initial contour. The contour deformation is iterated to get the final output

As the central module of the AID workflow, the segmentation network should be able to learn accurate segmentation from a small number of training images and a few AID iterations. To achieve this objective, we take advantage of the strong organ shape prior embodied in the organ contour representation, as inspired by the recently proposed contour-prediction models [26–28] in the computer vision field. We develop a 2D contour generation network that first generates an initial contour surrounding the target organ and then deforms the contour to fit the organ boundary. Figure 3 illustrates the architecture of this network. An advantage of this model is that it produces plausible organ shapes even when the contour fitting accuracy is imperfect at the preliminary training stage. This feature greatly eased the proofreading operation since the user simply needs to adjust the local contour position without modifying its topology.

For contour initialization, we used a network structure similar to CenterNet [29] to generate the bounding boxes of the target organs. However, the original CenterNet model conducts eight times downsampling to generate the final feature map. Although this strategy facilitates efficient computation for natural images, it reduces the segmentation accuracy for medical images. To tackle this problem, we propose a new backbone network with multi-scale feature upsampling and concatenation. The structure of the new backbone is shown in Fig. 3, where the circles denote convolution and the numbers in the circles represent the downsampling scale. To preserve fine-scale boundary features, the downsampled feature maps are upsampled and concatenated with the same-scale feature maps before downsampling, resulting in more accurate representation of the organ boundaries.

After the generation of the bounding box, the midpoints of the four box edges are connected to form a diamond-shaped initial contour, with N vertices evenly sampled along the contour. The CenterNet contains a deep layer aggression (DLA) CNN [30] for feature map calculation and a regression part for bounding box generation. The outputs of the CenterNet includes the centre coordinate, the height and width of the bounding box and the class labels of detected objects. When there are multiple targets in one slice, their contours are initialized and detected simultaneously. We notice that misclassification of organ types happens when the appearance of one organ resembles that of another. For example, in the slices that pass the lower liver, the liver may appear like the spleen. This issue is easily tackled by correcting the class label according to the bounding box position in the image. We also set the upper, middle and lower parts of the liver as three different classes. This helps to reduce intra-class diversity and avoid misclassification.

The multi-scale feature extraction backbone produces 128-channel feature maps. For a contour vertex $i$ with coordinate ${{\textbf{x}}_{i}} = \left( {x_{i} ,y_{i} } \right)$, its feature is represented as $f_{i} = \left( {F\left( {{\textbf{x}}_{i} } \right),x_{i}^{\prime } ,y_{i}^{\prime } } \right)$, where $F\left( {{\textbf{x}}_{i} } \right)$ is a 128-dimensional feature vector which is bilinearly interpolated from the CNN backbone feature map with the coordinate $x_{i}$. $\left( {x_{i}^{\prime } ,y_{i}^{\prime } } \right)$ is a translation-invariant vertex coordinate that describes the relative position of the vertex $i$ in the whole contour. $\left( {x_{i}^{\prime } ,y_{i}^{\prime } } \right)$ is calculated by subtracting $\left( {\min _{i} \left( {x_{i} } \right),\min _{i} \left( {y_{i} } \right)} \right)$ from $\left( {x_{i} ,y_{i} } \right)$, i.e. $\left( {x_{i}^{\prime } ,y_{i}^{\prime } } \right) = \left( {x_{i} ,y_{i} } \right) - \left( {\min _{i} \left( {x_{i} } \right),\min _{i} \left( {y_{i} } \right)} \right)$. The combination of $\left( {x_{i}^{\prime } ,y_{i}^{\prime } } \right)$ into the vertex feature implants the shape memory of the training set into our DL model.

The contour deformation network is a one-dimensional (1D) CNN that takes $f_{i}$ of all *N* vertices as the input. Since the contour is a closed polygon, features of the *N* vertices are treated as 1D periodic discrete signal and the circular convolution [28] is used instead of the standard 1D convolution, i.e. the convolution result of each vertex is calculated using the features of its left and right neighbours. We use a fixed length (*l* = 9) of the circular convolution kernel. The circular convolution layer is combined with a batch normalization layer and a rectified linear unit (ReLu) layer to form a convolution block. The network contains a concatenation of eight such convolution blocks with residual links between adjacent blocks. The outputs of all blocks are concatenated as the multi-resolution feature vector containing the features of different scales. Finally, three 1 × 1 convolution layers are applied to regress the 2D deformation vectors of the vertices, and the deformation vectors are added to the vertexes coordinates to obtain the deformed vertex locations. The above feature interpolation and deformation vector regression procedures are repeated *k* times to let the contour vertices gradually converge to the organ borders. In this study, empirical values of *N* = 128 and *k* = 3 are used.

The contour deformation network is trained using the same loss function as defined in [28] containing the positional losses of both the extreme points (${\textbf{L}}_{{{\text{ext}}}}$) and the contour vertices (${\textbf{L}}_{{{\text{cont}}}}$),

1 $$  \begin{array}{*{20}l} {{\textbf{L}} = {\textbf{L}}_{{{\text{ext}}}} + {\textbf{L}}_{{{\text{cont}}}} = \frac{1}{4}\mathop \sum \limits_{i = 1}^{4} {\textbf{l}}_{1} \left( {{\textbf{x}}_{i}^{{{\text{ext}}}} - {\textbf{p}}_{i}^{{{\text{ext}}}} } \right) }\\\qquad\quad{+ \frac{1}{N}\mathop \sum \limits_{j = 1}^{N} {\textbf{l}}_{1} \left( {{\textbf{x}}_{j}^{{{\text{cont}}}} - {\textbf{p}}_{j}^{{{\text{cont}}}} } \right)} \\ \end{array} $$

where ${\textbf{x}}_{i}^{{{\text{ext}}}}$ s are four contour vertices corresponding to four extreme points of the initial diamond-shaped contour. ${\textbf{x}}_{j}^{{{\text{cont}}}}$ s are the *N* vertices of the contour. ${\textbf{l}}_{1}$ denotes the smooth norm defined in [31]. The ground truth contours of the training data are generated from the expert-labelled organ regions. The upper, lower, left and right extreme points of the ground truth contour are obtained as ${\varvec{p}}_{j}^{{{\text{ext}}}}$, and *N*/4–1 vertices are evenly sampled along the contour between each pair of extreme points so that *N* vertices (i.e. ${\varvec{p}}_{j}^{{{\text{cont}}}}$) including the extreme points are obtained. ${\textbf{L}}_{{{\text{ext}}}}$ guarantees the positional correspondence of the extreme points of the deformed contour, ${\textbf{L}}_{{{\text{cont}}}}$ regularizes the edge distance.

In our application, a single contour deformation network is used for multiple organs. Thanks to the inclusion of translation-invariant coordinates in the vertex features, the network memorizes the contour shape of different organs at different slices. Circular convolutions at multiple resolution scales help to capture the global contour shape and guarantee the plausibility of the generated contour shape.

### Contour-based boundary adjustment

The amount of human labour spent at the proofreading stage not only depends on the accuracy of automated annotation but also relies on the proofreading method. Since the automated generated contour shape is guaranteed to be anatomically plausible, direct boundary adjustment with surface dragging can considerably save the proofreading time. We realize a real-time surface editing tool (as shown in the supplementary video) based on free-form deformation (FFD) [32] which is rarely provided in the existing open-source tools.

**Fig. 4:**
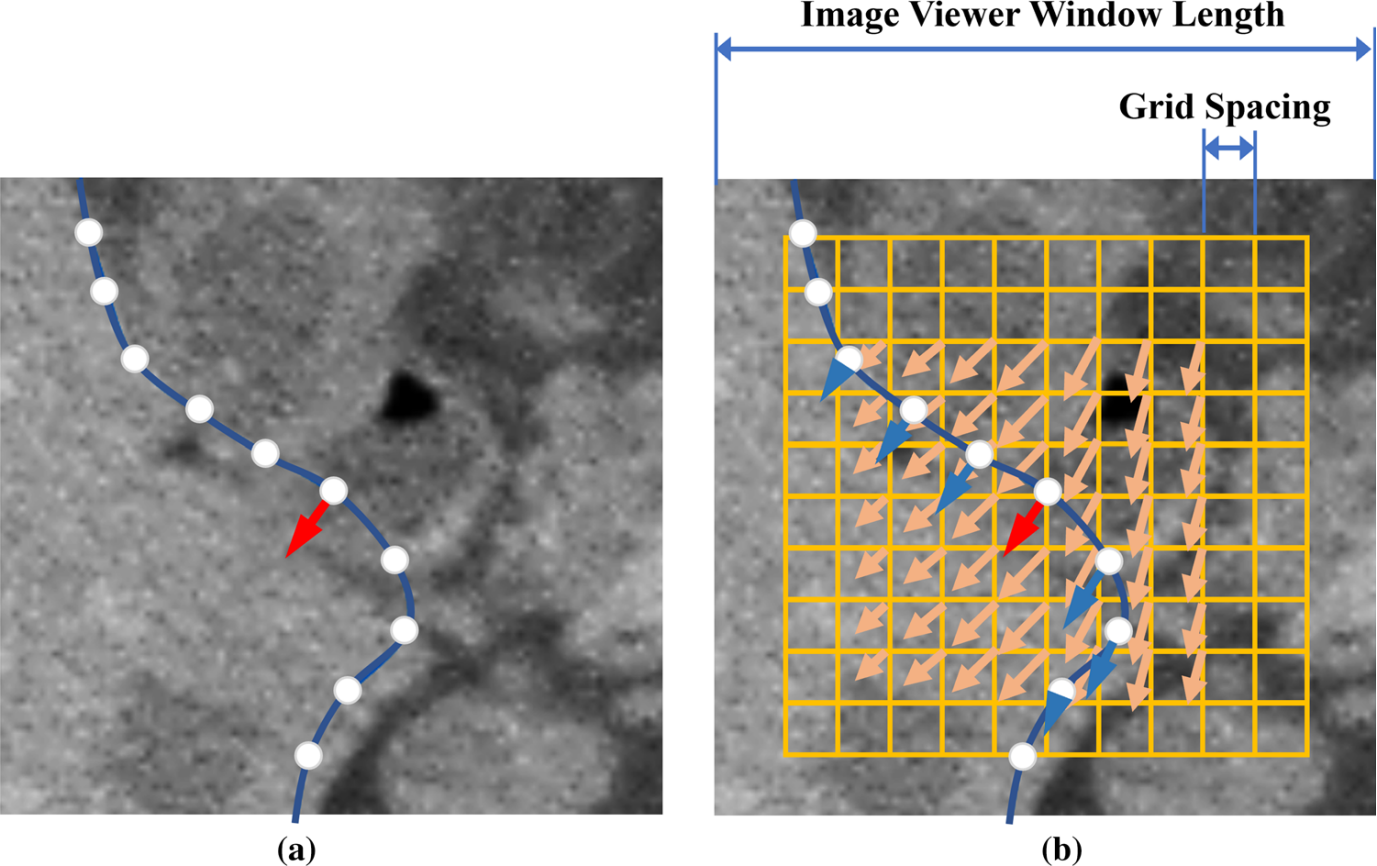
FFD-based interactive contour adjustment. **a** The user applies a translation vector (red arrow) to a point of the contour (blue curve) via mouse or stylus dragging. **b** Deformation vectors (orange vectors) of the FFD control grid (yellow grid) are calculated from the user input, and the deformation vectors of the contour vertices (blue arrows) are interpolated from the control grid deformation vectors

The proofreading starts from the adjustment of automatically generated 2D contours. As shown in Fig. 4, when the user starts to drag a point on the contour, a *k* × *k* control grid centred at the dragging point is generated. The spacing between the grid nodes is set to 1/(*k* + 1) of the length of the image viewer window. Therefore, the user can intuitively zoom the images in the viewer window to adjust the image area affected by the control grid. In this work, we use *k* = 10 works for all the test images.

The dragging event creates a deformation vector at the dragging point (red arrow in Fig. 4a and b). Using this deformation vector, the deformation vectors of all the grid nodes (orange arrows in Fig. 4b) are calculated by solving an inverse-interpolation problem [33]. We only computed the deformation vectors of the central (k−4) × (k−4) grid nodes and force the deformation vectors at the peripheral nodes to zero. Then, the deformation vectors of all the contour vertices (blue arrows in Fig. 4b) are interpolated using the B-spline polynomial function,

2 $$ \begin{array}{*{20}c} {{ \textbf{v}} = \mathop \sum \limits_{i = 0}^{m} \left( {\begin{array}{*{20}c} m \\ i \\ \end{array} } \right)\left( {1 - u} \right)^{m - i} u^{i} \left( {\mathop \sum \limits_{j = 0}^{n} \left( {\begin{array}{*{20}c} n \\ j \\ \end{array} } \right) \left( {1 - v} \right)^{n - j} v^{j}  {\textbf{v}}_{i,j} } \right)} \\ \end{array} $$

where $\textbf{v}$ is the interpolated deformation vector of a contour vertex, (*u*, *v*) is the normalized local coordinate of ${\textbf{v}}$ in the control grid coordinate system, $v_{i,j}$ is the deformation vector of grid node (*i*, *j*), *m* and *n* are the numbers of grid nodes used for the interpolation. We use *m* = *n* = 3 in this study, i.e. the local 4 × 4 grid around the interpolated vertex is used for the interpolation.

The FFD adjustment is applied to the automated generated contours in a slice-by-slice manner. The user does not need to proofread every slice since the contour of adjacent slices are usually similar. The user may selectively adjust several parallel slices and we provide a contour interpolation method to automatically generate the contours of the skipped slices. The contours of the adjusted slices are converted into 2D level set maps (signed distance functions, SDFs) and a second-order polynomial interpolation is applied to interpolate the level set maps of the skipped slices. Finally, the binary maps of all the slices are obtained by thresholding the SDFs and are stacked to create a binary label volume of the segmented organ.

Usually, the label volume generated as above is accurate enough for proofreading purposes. For organs with complex shapes (e.g. the liver and spleen), additional adjustments are sometimes needed in the slices orthogonal to the generated contours. We convert the binary label volume into a triangular surface mesh using the marching cube method [34] and extend the 2D FFD adjustment method to 3D for the adjustment of the surface mesh. The 3D version of the B-spline polynomial function is a trivial extension of Eq. (2) as described in [32]. The 3D FFD method facilitates easy manipulation of the annotation result in 3D space.

## Results

For validation, our method is compared with two approaches widely adopted by the research community, i.e. manual annotation assisted by inter-slice interpolation and the AID method based on voxel-label representation.

The first method is mostly used for segmentation network training and is implemented in the MITK software [13]. With this method, the user selectively annotated the organ contours in a few parallel slices and then performed contour interpolation to obtain the contours in all slices. This method is similar to the selective contour annotation step of our method except that the contours are manually created. We compare our method with this method to evaluate the influence of the contour-learning network on inter-operator consistency and proofreading efficiency. For both methods, the number of selected slices was determined by the human operator to ensure accurate annotation.

The second method follows the similar AID workflow of our method. The main difference is that it uses the conventional voxel-label prediction model for automated segmentation. The prediction model is a Dense V-net [35], and the loss function is the weighted sum of an L2 regularization loss with label smoothed probabilistic Dice scores for each organ. We choose this method for comparison to prove the advantage of contour-based representation on shape learning and human proofreading. For a fair comparison, we used the same human-proofreading scheme (i.e. our contour correction method) for both methods.

The comparison was made from the aspects of annotation accuracy, network learning ability, inter-rater stability and proofreading efficiency based on a multi-institute abdominal CT image dataset. The dataset includes 20 CT volumes with pixel sizes between 0.72 and 1.37 mm and slice spacing between 1.60 and 3.00 mm, consisting of four images from the online 3D-irCadb database^1^ and 16 retrospective images from four of our collaborated hospitals across the country. The images were acquired with 28–298 mA tube current and 100–140 kV tube potential. All the images had corresponding label images of the liver, spleen and kidneys annotated by an experienced human expert as the ground truth.

Two well-trained human operators were invited to independently use the three methods to annotate the 20 volumetric images slice by slice. For each operator, the Dice scores and the averaged symmetric surface distances (ASDs) were computed using the organ labels of the experienced expert as the ground truth. When comparing different methods, we averaged the Dice scores and ASDs of the two operators for each image and used the averaged value for method-wise comparison. When evaluating inter-operator variabilities, we ignored the labels of the experienced expert and computed the mutual Dice and ASD between the two operators to measure their discrepancies.

For the validation of the two AID-type methods (i.e. our method and the voxel-label-based AID), a fourfold cross-validation strategy was used. The 20 CT volumes were randomly divided into fourfolds. Each time onefold was used as the test images and the other threefolds were used for network training. From the threefolds of training data, onefold was randomly selected to train a preliminary network which was then used to automatically annotate the other twofolds of training data. From each training volume image, all the 2D slices were used for training the contour-based network. The human operators proofread the annotations of the preliminary network and retrained the network using the threefolds of training data. Our method was trained end-to-end for 300 epochs. The learning rate started from 1e−4 and decayed by half at 80, 120, 150 and 170 epochs. The Dense V-net was trained with a learning rate of 0.01 and a batch size of 6 for 200 epochs.

**Fig. 5:**
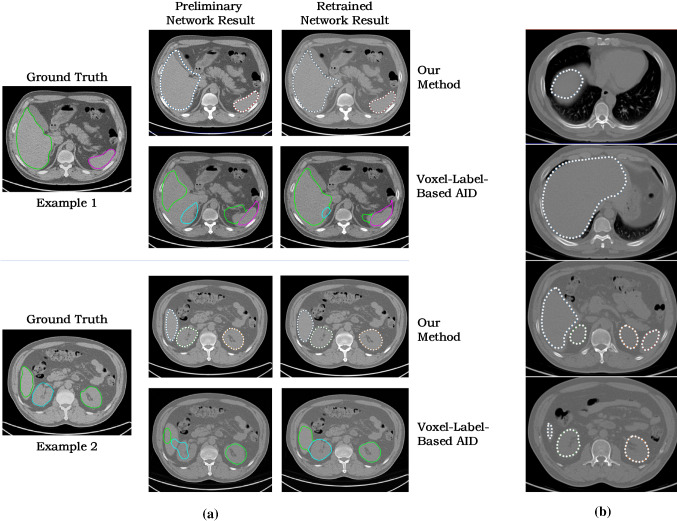
Visual observation of organ segmentation results. **a** Representative results of two different slices, using the preliminary network and the retrained network of our method and the voxel-label-based AID method, respectively. The contour lines with control points and coloured circles in the figure represent the segmentation results of the different organs. **b** The segmentation results of our method in the slices of different vertical levels

For visual inspection, Fig. 5a shows the automated annotation results of the two AID-based methods in two representative slices. For both methods, the retrained network yielded more accurate results than the preliminary network. It is encouraging to see that the results of our preliminary network maintain plausible organ shape although the contours do not perfectly fit the true organ boundary. In contrast, the preliminarily trained voxel-label-based network produces erroneous contour shapes due to insufficient training data. Figure 5b displays the segmentation results in 2D slices of different vertical levels, including the middle, transition and periphery slices of the organs.

**Fig. 6:**
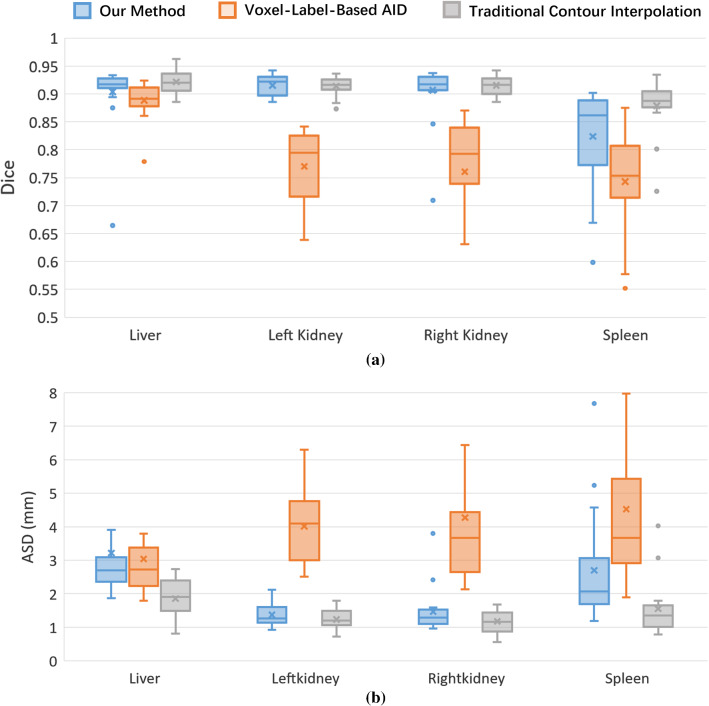
Box plots of **a** Dice and **b** ASD of the three methods for different organs

As a quantitative comparison of the annotation accuracy, Fig. 6 shows the box plots of Dice and ASD of the three methods. For the traditional method, Dice and ASD were calculated for the contour interpolation results. For the two AID-based methods, Dice and ASD were calculated for the automatic annotation results of the retrained network. The traditional contour interpolation achieved the highest median Dice (> 0.88) and lowest median ASD (< 2.0 mm) for all the organs. This is because the annotations of the traditional method were almost generated manually. In contrast, the annotations of the two AID-based methods were generated by the network model; therefore, were less accurate than the traditional method. Nevertheless, we can see that our method obtained a comparable accuracy (median Dice > 0.85 and median ASD < 2.5 mm) to the traditional methods with only two iterations. The voxel-label-based AID method was the least accurate. The boxes of this method were also the widest among the three, implying imperfect robustness against different test images.

**Fig. 7:**
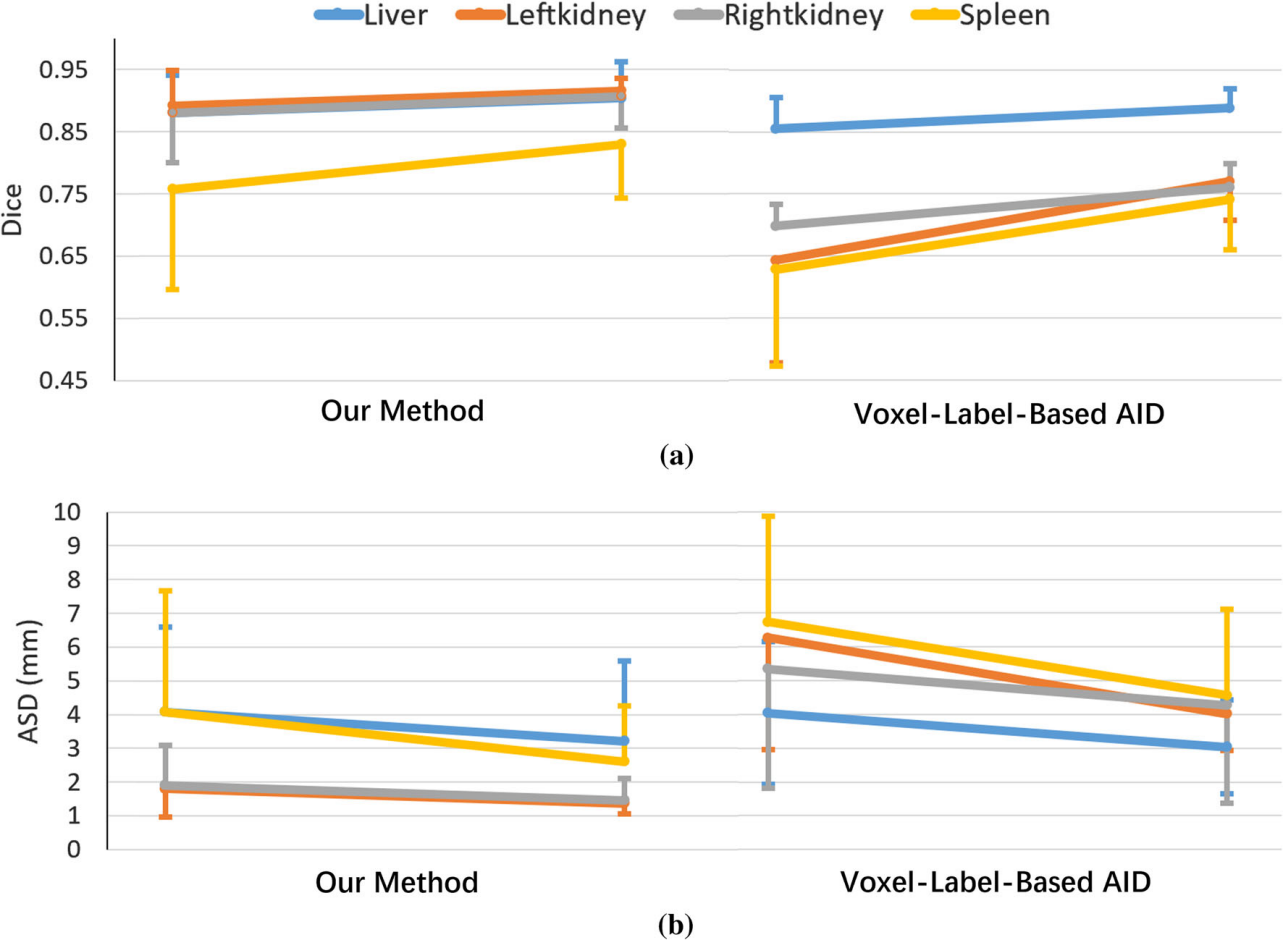
Comparison of **a** Dice and **b** ASD before and after the retraining step, between our method and the voxel-label-based AID method. For each organ, the left and right ends of the oblique line show the mean and std. of the preliminary network and the retrained network, respectively. For figure clarity, the standard deviation bars of different organs are plotted in different directions

The learning ability comparison was made between our method and the voxel-label-based AID method. Figure 7 shows the mean and standard deviation values of Dice and ASD before and after the retraining. After the retraining, accuracy improvements (i.e. higher mean Dice with smaller standard deviations) are observed for both methods. Our method was more accurate than the voxel-label-based AID method either before or after the retraining. For the kidneys and the spleen, our preliminary network is even more accurate than the retrained Dense V-net, thanks to the shape knowledge learned from the very small preliminary training dataset.

**Fig. 8:**
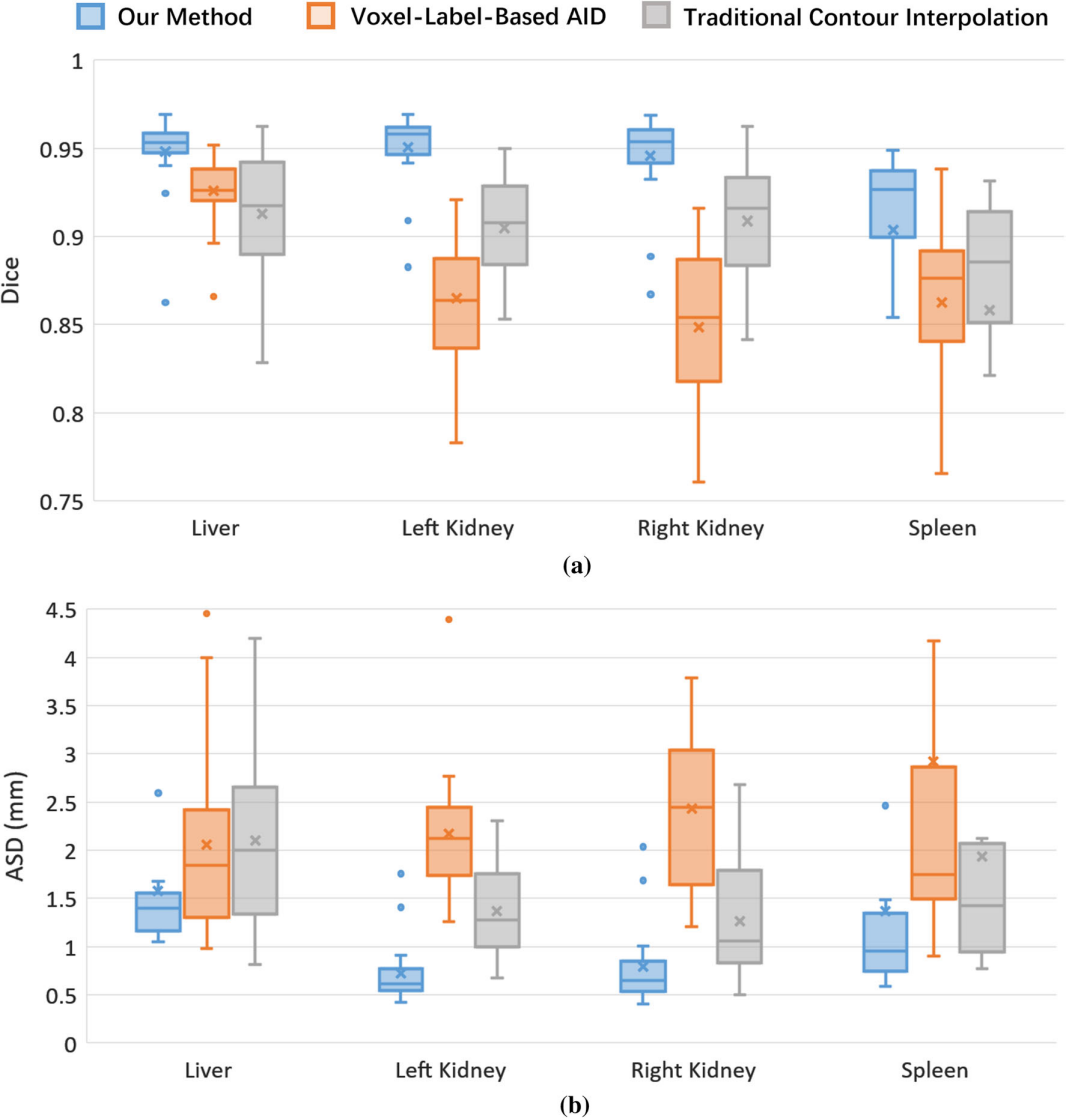
Inter-operator **a** Dice and **b** ASD box plots of the three methods for different organs

To compare the inter-rater variability, the mutual Dice and ASD between the two operators were calculated and are plotted in Fig. 8. Our method demonstrates the highest median Dice (> 0.93) among the three methods, meaning the best inter-operator agreement for the annotated organ regions. The inter-operator ASDs of our method was not the lowest but almost approached the most accurate one (the traditional method). The traditional method is the most accurate in terms of the contour distance (ASD) because its contours were manually sketched by the human operators. Nevertheless, the boxes of our method are much narrower than the other two methods, meaning that our method is superior in inter-rater consistency since our contour-correction method reduces the amount of subjective interaction.

**Fig. 9:**
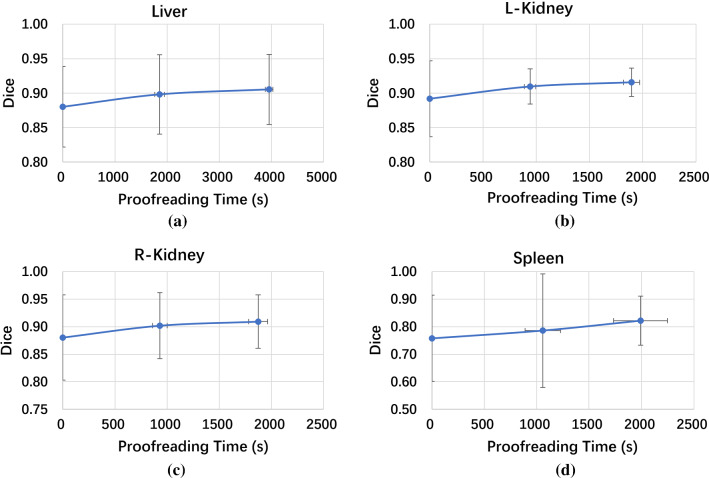
Dice curves versus proofreading time for **a** the liver, **b** left kidney, **c** right kidney and **d** spleen

To evaluate the annotation efficiency, the operators recorded the interaction time required for proofreading the automatic annotation results of the preliminary network. The proofreading time per image per iteration was 16.19 ± 2.58 min for our method and 29.79 ± 3.50 min for the voxel-label-based AID method. Our method requires much less interaction time because our preliminary network is more accurate and reasonable than Dense V-net. Figure 9 shows the multi-iteration Dice increment curves with increasing proofreading time. As the proofreading time increases, the performance of the network continues to get better. For most organs, the Dice score reaches 0.9 within 20 min.

To further validate the few-sample learning ability, we compared our contour prediction network with the state-of-the-art (SOTA) nnU-Net [36] based on three additional datasets. The first dataset is the open-source Chest X-ray Masks and Labels [37, 38], containing 704 chest X-ray images with lung area annotations. The second dataset includes 88 cardiac ultrasound images from a local hospital with myocardium annotations by experienced physicians. The third dataset is the Lung Tumours dataset of the medical segmentation decathlon [39] and contains 64 CT volumes with tumour area annotations.

**Fig. 10:**
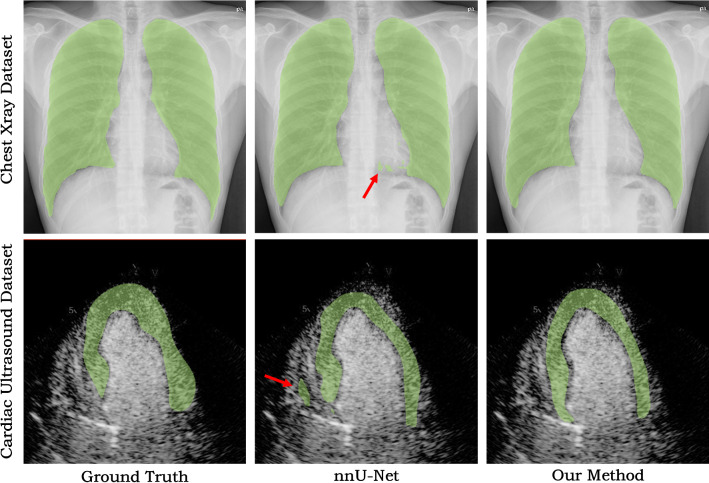
Representative segmentation results of few-sample learning, comparing our network and nnU-Net based on two different datasets. The red arrow shows the topology error

For the first two 2D datasets, we randomly selected a small number of 2D training slices (21 and 66 images for the X-ray and ultrasound datasets, respectively) and validated the trained model on the rest of the datasets as testing images. Due to the limited number of training data, both methods produced minor segmentation errors in some test images, as shown in Fig. 10. However, nnU-net tends to generate topological errors (pointed by red arrows) which needs tedious manual correction. In contrast, our approach consistently yields anatomically plausible shapes which are easy to correct with our contour dragging tool.

**Fig. 11:**
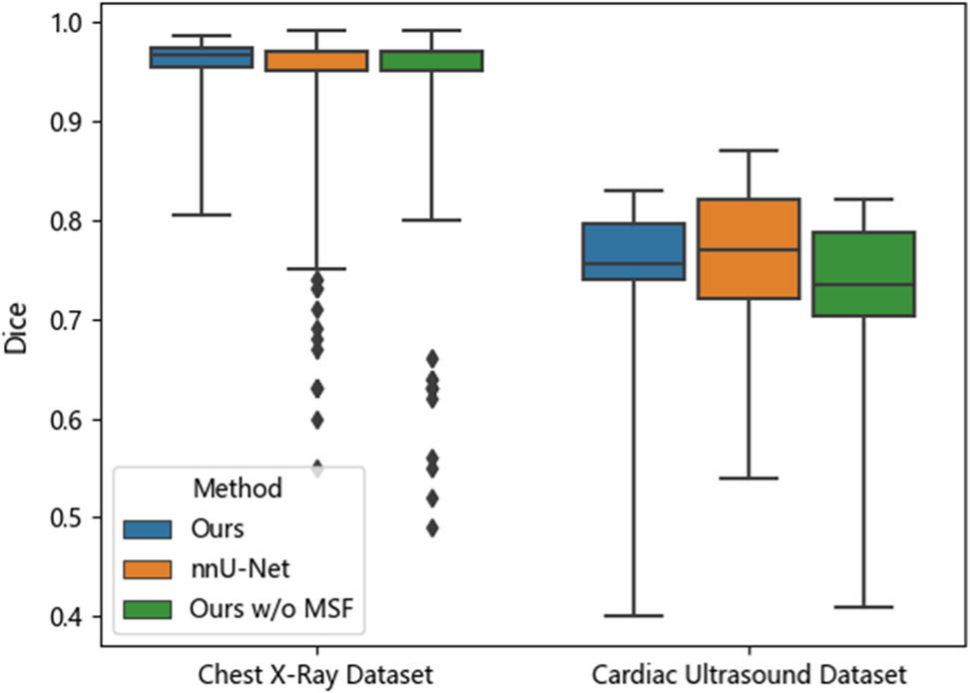
Dice of our network, nnU-Net and our network without the multi-scale feature (MSF) extraction backbone

As a quantitative comparison, box plots of Dice scores of both networks are shown in Fig. 11. We also added another compared network which used the same architecture of ours but replaced the multi-scale feature (MSF) extraction backbone with the conventional CenterNet backbone (named ‘ours w/o MSF’). For both datasets, our method achieved a median Dice close to that of nnU-Net, with the guarantee of correct topology. The boxes of our method were also narrower than that of nnU-Net, implying better robustness against different test images. Compared to ‘our network w/o multi-scale feature’, our network also achieved more accurate and more robust results, proving the advantage of the multi-scale feature extraction backbone. Besides, the inference time of our method is much shorter than nnU-Net (both using one NVIDIA RTX 3090 GPU). On the chest X-ray dataset, the inference time per image was 26.32 ± 6.22 ms for our method and 4698.51 ± 1124.65 ms for nnU-Net. On the cardiac ultrasound dataset, the inference time per image for our method and nnU-Net was 19.93 ± 1.26 ms and 181.63 ± 11.62 ms, respectively. The reasons for the significant time advantage of our network are attributed to the simpler network structure and smaller parameter size, as well as the avoidance of using sliding convolution kernel and multiple mirror inference. Faster inference helps to reduce device dependency and is more beneficial for real-time applications such as cardiac ultrasound detection.

We further validated the 3D segmentation performance of the networks with the lung tumour segmentation dataset [39]. Compared to 2D segmentation networks, the 3D segmentation network faces more severe challenges of larger network parameter size and less training data. Because our method is based on contour representation, it is more light-weight than the voxel-based CNNs; thus, it suffers less from the problem of large parameter vs. small training set. Moreover, our method is able to learn plausible contour shape from small training set. Without the shape prior, nnU-Net may yield unsatisfactory segmentation with insufficient training dataset. In addition, our method is also compared with the nnU-Net with boundary loss [40] for segmenting small objects. Different from our contour-based learning method, this boundary loss approximates the differential boundary variation using an integral approach, which avoids completely local differential computations involving contour points and represents boundary change as a regional integral. In this way, the boundary loss is easily combined with the standard regional losses, such as Dice or cross-entropy. The boundary loss improves the performance for highly unbalanced segmentation tasks in which the voxel number differs by several orders of magnitude across classes. In the experiment, a fourfold cross-validation strategy was used. The 64 volumetric test images were evenly divided into fourfolds. Each time onefold (16 images) was used as the training images, and the other threefolds (48 images) were used for testing to simulate the small training data situation. The nnU-Net with boundary loss is trained following the training method in [40].

**Fig. 12:**
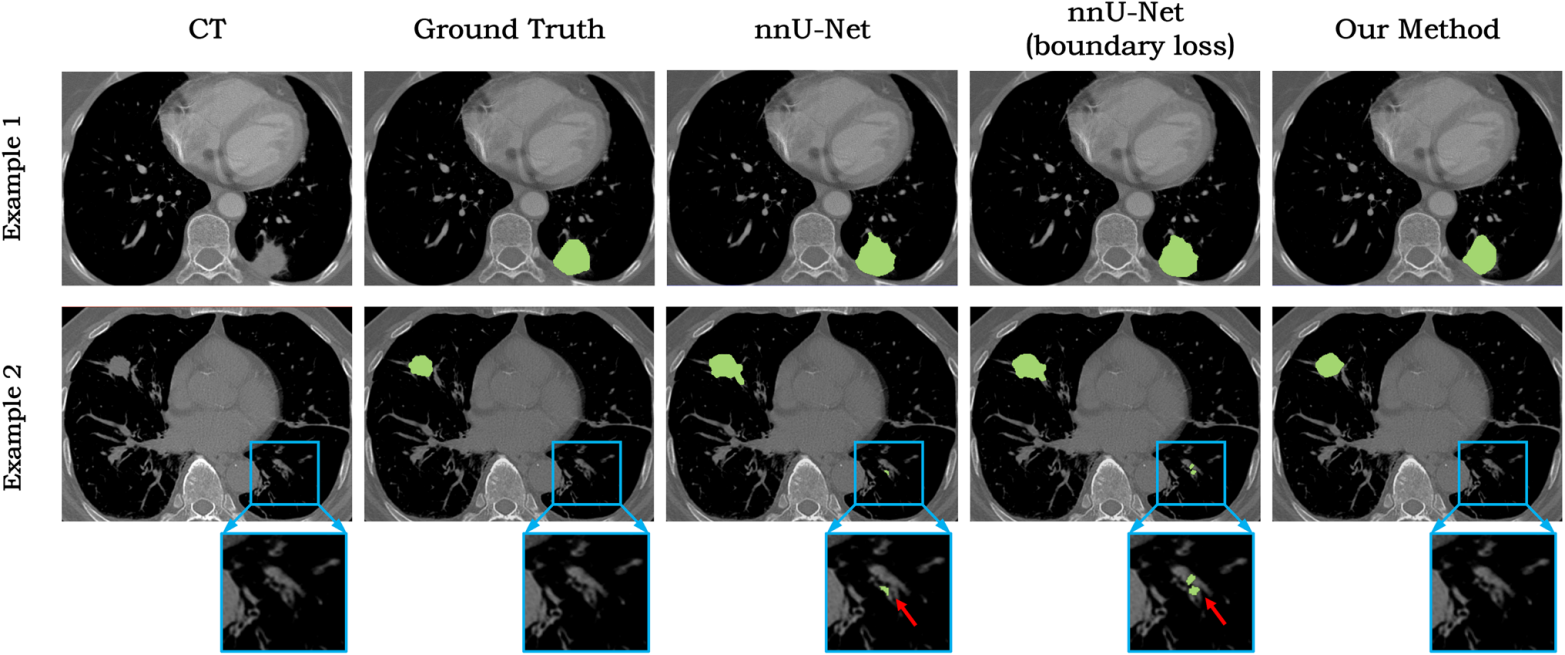
3D lung tumour segmentation comparison between our method and nnU-Net with/without the boundary loss. The blue squares display the zoomed area, and the red arrows point to the false positive segmentation

Figure 12 shows two representative results of the comparison experiment. With insufficient training data, nnU-Net yielded unsmooth tumour boundary for example 1 and false positive segmentation of the sporadic vessels for example 2. With the boundary loss, the boundary smoothness was improved, but the false positives still exist. Our method generates smooth segmentation and avoids the false positives of implausible tumour shape, thanks to the ability of shape learning from limited training data.

**Fig. 13:**
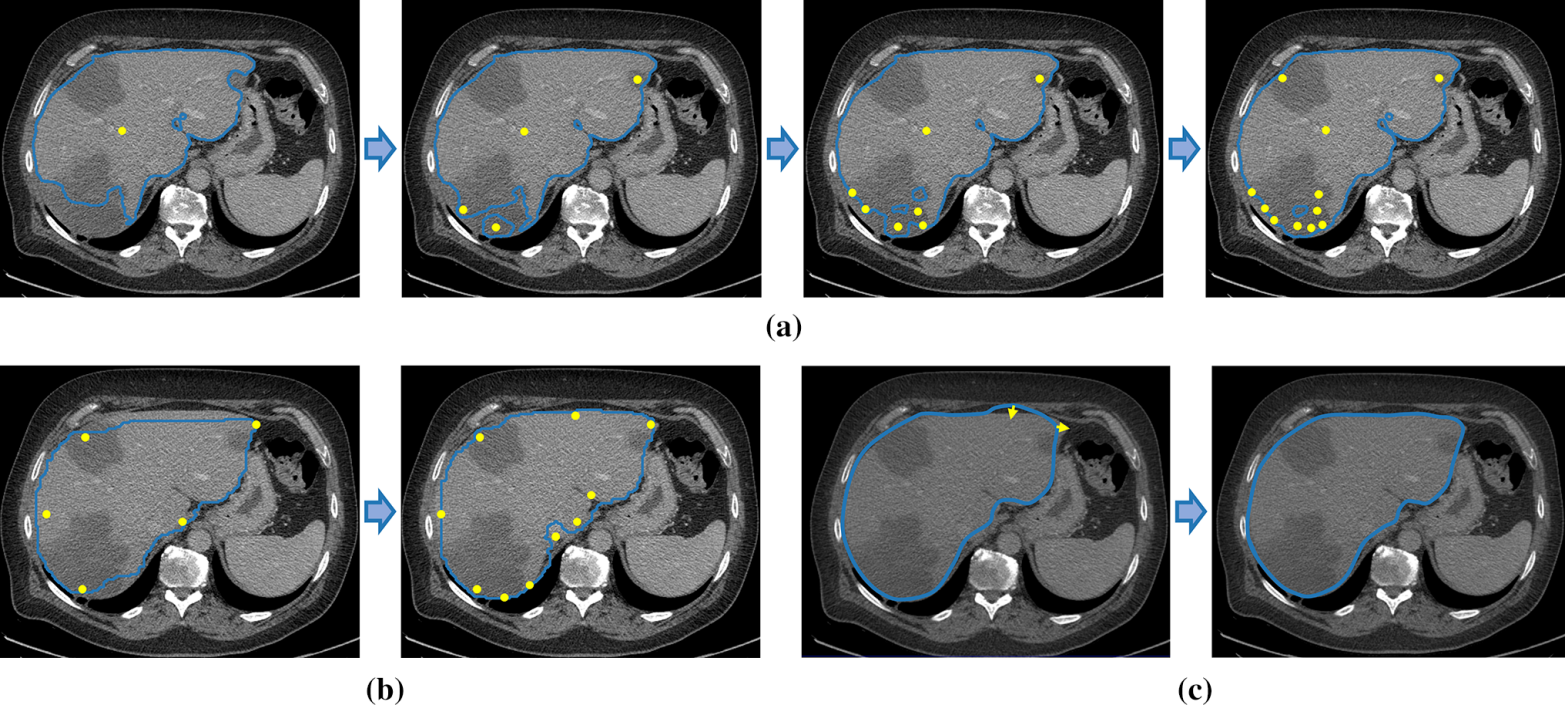
Representative proofreading process of **a** MIDeepSeg, **b** DeepGrow and **c** our method. In each subfigure, the user-interaction process is denoted by sequential frames connected by the blue arrows indicating the sequence order. The blue contour lines indicate the segmentation results. The yellow points in **a** and **b** are foreground seed points. The yellow arrows in **c** indicate the user’s dragging operation

To evaluate the efficiency of the contour-based boundary adjustment, we compared it with two deep-learning-based interactive segmentation mothed, i.e. MIDeepSeg [41] and DeepGrow [24]. Both methods learn the way to react to user-input seed points, while MIDeepSeg is trained with images of multiple modalities and target objects for generic segmentation tasks, and DeepGrow needs to be specifically trained for the target modality and object. For MIDeepSeg, we used the implementation provided by authors. For the DeepGrow model, we used a model trained for abdominal CT images.^2^ We chose a difficult task of segmenting tumour-contaminated liver. As shown in Fig. 13, although the two compared models were well trained for the interaction scenario, they need many user inputs at the difficult regions (e.g. big tumour region and fuzzy liver boundary) and finally did not converge perfectly. This is mainly because that tumour appearance is highly variable between patients, thus these methods perform unsatisfactorily for unseen tumours. In contrast, our method only needs two simple drags because the contour network learned strong shape *prior* of the liver. Of course, our method is mainly developed for the objects with shape priors, while the two compared methods are not restricted to the segmentation of regular shape.

## Discussions

As analysed in the Introduction section, the efficiency of an AID workflow is directly affected by the learning ability of the DL model. We focus on developing a DL model which only needs a few training images and a few AID iterations to achieve satisfactory annotation accuracy. Experimental results show that our contour prediction network achieved a mean Dice over 0.86 for the liver and kidneys using only five preliminary training images. Such a level of accuracy is even better than the retrained Dense V-net. After retraining our network with 10 more images, the median Dice scores of the liver and kidneys were above 0.92, and the median ASDs of the kidneys and spleen drop below 1.5 mm. Thanks to the good learning ability from limited training data, the human operators only need to make very few corrections in the proofreading step; therefore, our method requires much less proofreading time than the voxel-label-based AID method. A human operator using our method only took ~ 16 min for each training image, i.e. ~ 4 min per organ.

The good learning ability of our method is attributed to the use of contour-based shape representation instead of the voxel-label representation. It should be mentioned that boundary information has been used in recent segmentation networks, such as the boundary loss for improving small object segmentation [40]. Different from the loss-based methods, our method represents boundaries with contour points, facilitating strong shape memory and efficient boundary proofreading. The combination of contour point coordinates into the vertex feature vector implants the memory of contour shape into the network, helping the network to produce plausible contour shape even when the training images are not abundant. The short proofreading time of our method is attributed to the contour-based adjustment method. Since the automatically generated contours are already close to the true boundaries, the efforts required for contour adjustment are much reduced. Moreover, our interactive proofreading method facilitates convenient contour correction via simple mouse dragging, saving considerable interaction time as compared to the conventional voxel-label correction.

Another advantage of our method is low inter-operator variability. As reflected from the mutual Dice and ASD between the two human operators, our method demonstrates good agreements between the two operators. This is because the annotation was generated by the retrained network rather than pure manual sketching. Because our network already had reasonable accuracy after preliminary training, the human operators need not perform too much proofreading; thus, the subjective influence on proofreading was reduced.

We noticed that both our method and the voxel-label-based AID method have different levels of annotation accuracy for different organs. The spleen usually has lower Dice than the other organs, and the liver tends to have higher ASD. This is because both the liver and spleen have complex shapes and fuzzy boundaries with the adjacent soft tissues. However, the traditional contour interpolation method does not have such obvious inter-organ differences, because the human operators have full control of the annotation accuracy. Therefore, we remind the readers that although the AID-based methods save the annotation time, they may have variable accuracy for different organs.

The experimental results show that the voxel-label-based AID method needs more training data than our method to achieve satisfactory accuracy. However, voxel-label and boundary contour are like two sides of the coin. Although contour representation benefits the segmentation of regularly shaped objects (e.g. human organs), voxel-label may be more suitable for segmenting objects with complex or irregular shapes such as tumours. It should also be noted that the accuracy of our contour-based method may be constrained by the shape variability of the training set, yielding unsatisfactory segmentation of the organ shapes inexistent in the training data (e.g. lesion-distorted organ shape). Nevertheless, thanks to the FFD-based contour adjustment method, such inaccurate segmentation can be conveniently corrected with minor user interaction. For future work, we will make efforts on combining the advantages of contour-based on voxel-label-based method, as well as on improving the shape learning generalizability based on limited training samples.

## Conclusion

This paper introduces an iterative annotation and proofreading workflow to reduce the annotation workload for organ segmentation network training. By integrating a contour generation network and a convenient contour adjustment approach, organ annotation using our framework becomes more efficient, more accurate and less prone to inter-operator variability than using the voxel-label-based AID method and the traditional contour interpolation method. We have implemented this method as an extension model to our open-source software AnatomySketch.^3^ Our future work will explore better shape representation to improve the learning ability and generalizability for more complex objects such as tumours and vessels.

## Supplementary Information

Below is the link to the electronic supplementary material.

Supplementary file1 (WMV 957 kb)

## Data Availability

The source code and the proposed AID model are integrated into our open-source software AnatomySketch for the research community to use (https://github.com/DlutMedimgGroup/AnatomySketch-Software/tree/master/AID).

## Footnotes

1 3D-irCadb database: https://www.ircad.fr/research/3d-ircadb-01/.

2 https://www.medseg.ai/.

3 AnatomySketch: https://github.com/DlutMedimgGroup/AnatomySketch-Software.
